# Enhancing Facial Aesthetics Using a Novel Technique: A Case Report

**DOI:** 10.7759/cureus.64055

**Published:** 2024-07-08

**Authors:** Pooja Chitlange, Seema R Kambala, Tanvi Jaiswal, Surekha A Dubey

**Affiliations:** 1 Department of Prosthodontics and Crown and Bridge, Sharad Pawar Dental College, Datta Meghe Institute of Higher Education and Research, Wardha, IND

**Keywords:** complete denture, cheek plumper, aesthetics, rehabilitation, sunken cheek

## Abstract

A person's facial appearance matters in both social and professional contexts. Losing teeth has an impact on phonetics and mastication, but it also has an aesthetic effect on the lower face's appearance. Cheek sinking is caused by a decrease in muscular tone and an increase in the rate of resorption of the alveolar ridges. An edentulous patient will transform aesthetically if they receive facial as well as dental rehabilitation. The patient's social contacts and sense of self-worth both improve as a result. When an individual exhibits significant alveolar process resorption, traditional dentures are unable to offer sufficient support, hence requiring supplementary cheek support. Appliances for elevating or plumping the cheeks can be used for this. A 68-year-old male patient reported to the department of prosthodontics with a chief complaint of missing teeth and poor aesthetics. After a complete examination, a complete denture prosthesis with a detachable cheek plumper was planned. Press button retention for cheek plumpers is an advantage due to its easy installation and use. This article outlines a straightforward, inexpensive, and non-invasive process for creating a non-detachable cheek plumper using a press button for those who are edentulous. The two-in-one prosthesis enhances the appearance of the face while also replacing lost teeth.

## Introduction

Facial aesthetics are significant in both social and professional contexts. The loss of teeth affects not just phonetics and mastication but also the lower half of the patient's visual appearance [1]. Sunken cheeks are one of the many changes that aging brings about in the face. The loss of teeth results in a decline in muscular tone and an accelerated rate of residual alveolar ridge resorption, which both cause the cheeks to drop. A completely edentulous patient will transform aesthetically if they receive facial as well as dental rehabilitation. As a result, the patient's social interactions and self-confidence both rise [2]. The most popular method of treating an edentulous maxilla or mandible is a removable complete denture prosthesis. When an individual exhibits significant alveolar process resorption, traditional dentures are unable to offer sufficient support, hence requiring supplementary cheek support. Appliances for elevating or plumping the cheeks can be used for this [3].

Various methods of fabricating cheek plumpers are described in the literature. It can be broadly classified into two types, i.e., detachable cheek plumpers and non-detachable cheek plumpers. There are benefits to the detachable cheek plumper over the traditional, non-detachable ones. They are convenient, controllable, and comfortable to attach and remove. They are also simple to clean and avoid the muscle fatigue caused by prolonged use [4]. It is possible to incorporate attachments like press buttons, double die pins, and personalized attachments. Customized attachments require more work and extra stages in the lab. The patient needs to be careful while using a double die pin because if it is not inserted correctly, it can damage the mucosa. The magnet-retained cheek plumper's high magnetic force facilitates automatic reseating and ease of cleaning and placement, making it superior to other known techniques. Furthermore, smaller magnets are easily accommodated by the denture flange [5]. Every type of cheek plumper has its own merits and demerits. Undetachable cheek plumpers are standard prostheses in one piece with adjuncts on both sides of the buccal flanges of the denture, which are polished. Meanwhile, detachable cheek plumpers can be separated from dentures. This case report describes the unique method of fabrication of a detachable cheek plumper.

## Case presentation

A 68-year-old male patient visited the department of prosthodontics, primarily complaining of loss of teeth in both the arches for the past two months and wanting to get them replaced. The patient gave a history of extraction in the upper right and left-back regions of the jaw two months ago. The patient has been a mandibular denture wearer for two years. Upon intraoral inspection, the patient's maxillary and mandibular arches were completely edentulous, and the mandibular ridge was severely resorbed. A complete denture prosthesis was recommended to the patient after discussing all the available treatment options. A written consent was obtained from the patient.

**Figure 1:**
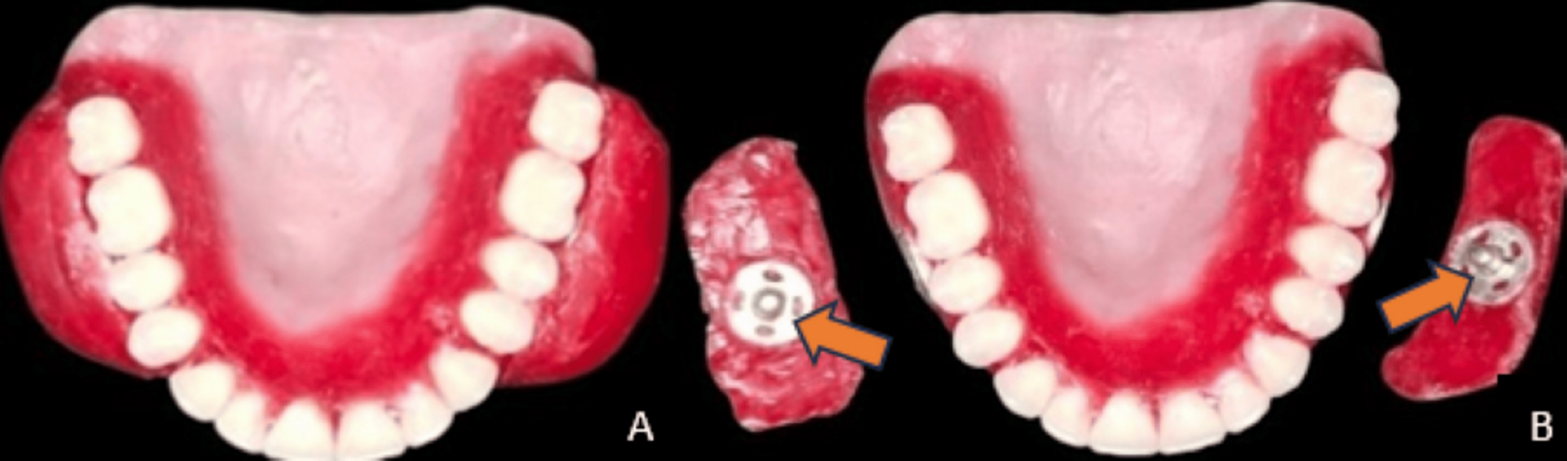
A: Complete denture prosthesis with cheek plumper; B: Complete denture prosthesis with detachable cheek plumper; arrow shows press button attachment for detachable cheek plumper to add bulk to complete denture prosthesis.

After obtaining the consent of the patient, preliminary impressions were made using an impression compound (Medical and Dental Market (MDM) Corporation, Delhi, India). Using tray material, a custom tray was fabricated on a preliminary cast (Dental Product of India (DPI) RR Cold Cure, Dental Products of India Ltd., Mumbai, India). Border molding was done using a low-fusion impression compound (DPI Pinnacle Tracing Sticks, Dental Products of India Ltd., Mumbai, India), and final impressions were made using zinc oxide eugenol impression paste (DPI Impression Paste, Dental Products of India Ltd., Mumbai, India). Denture base and wax rims were fabricated using cold cure resin (DPI Cold Cure, Dental Products of India Ltd., Mumbai, India) and modeling wax, respectively. Extra wax was added to the maxillary buccal flange's in the premolar and molar regions during jaw relation to create cheek plumper. After recording jaw relation, a mean-value articulator was used to mount the recorded jaw relation. The extraoral look verified the extent and proper support of the cheek plumper. During the jaw relation, the patient's preferences for comfort and appearance were also taken into account; a press button was added to make the cheek plumper detachable, as shown in Figure 1. The teeth were arranged in class I molar relation. After try-in, the site of cheek plumper and sufficient support were verified again. Denture processing was done following the try-in.

**Figure 2:**
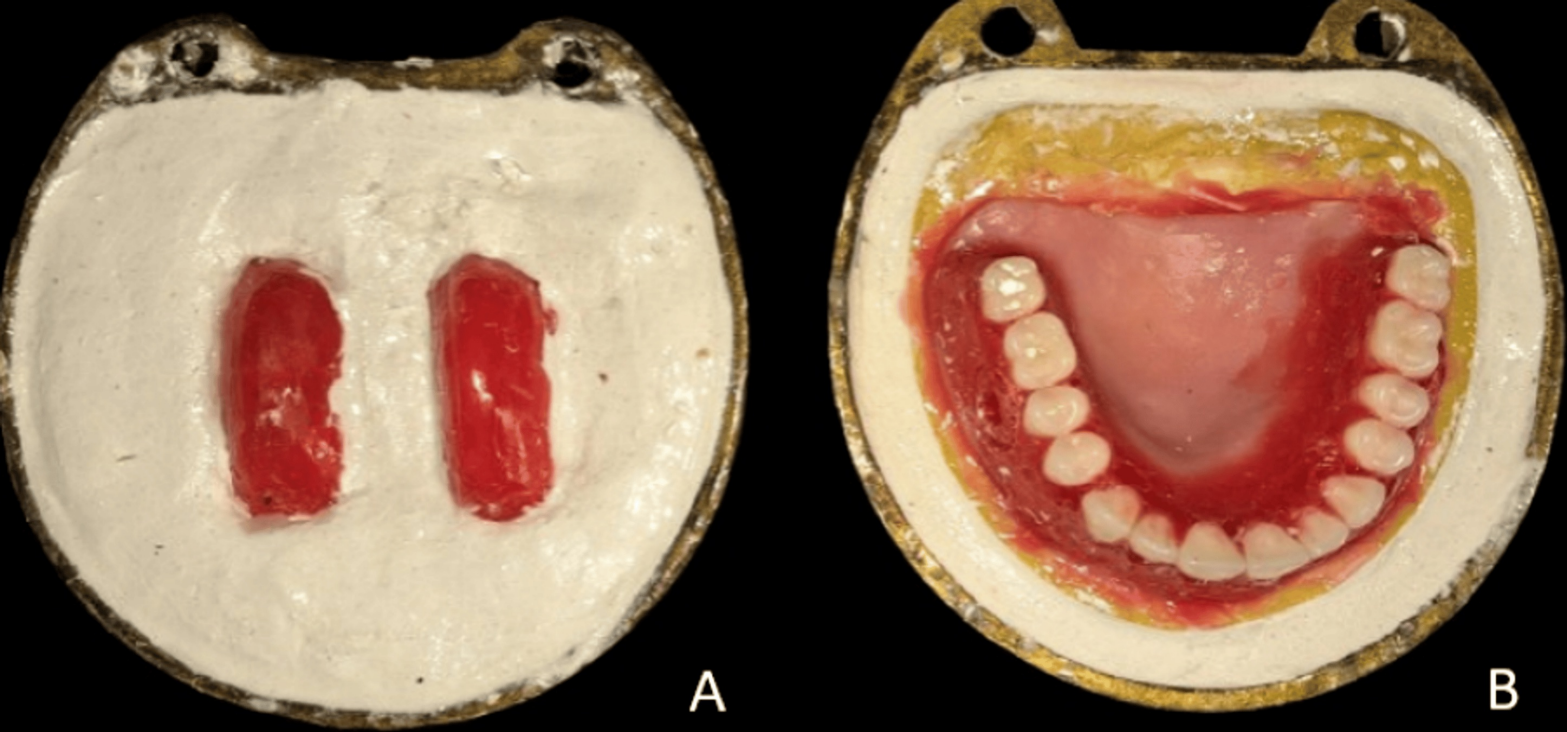
A: Processing of detachable cheek plumper; B: Processing of maxillary complete denture prosthesis.

**Figure 3:**
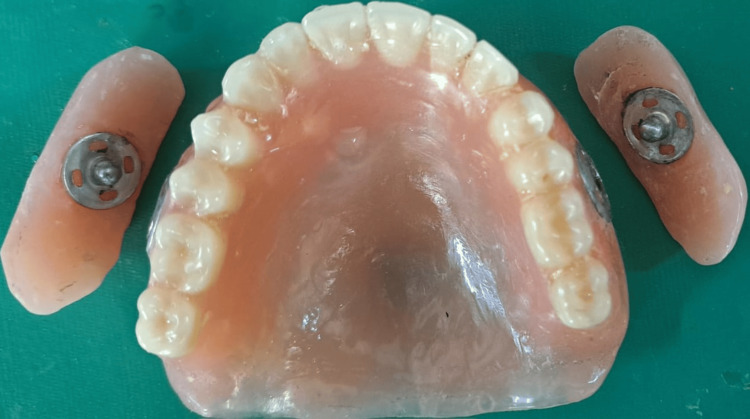
Finished and polished complete denture prosthesis with detachable cheek plumper.

**Figure 4:**
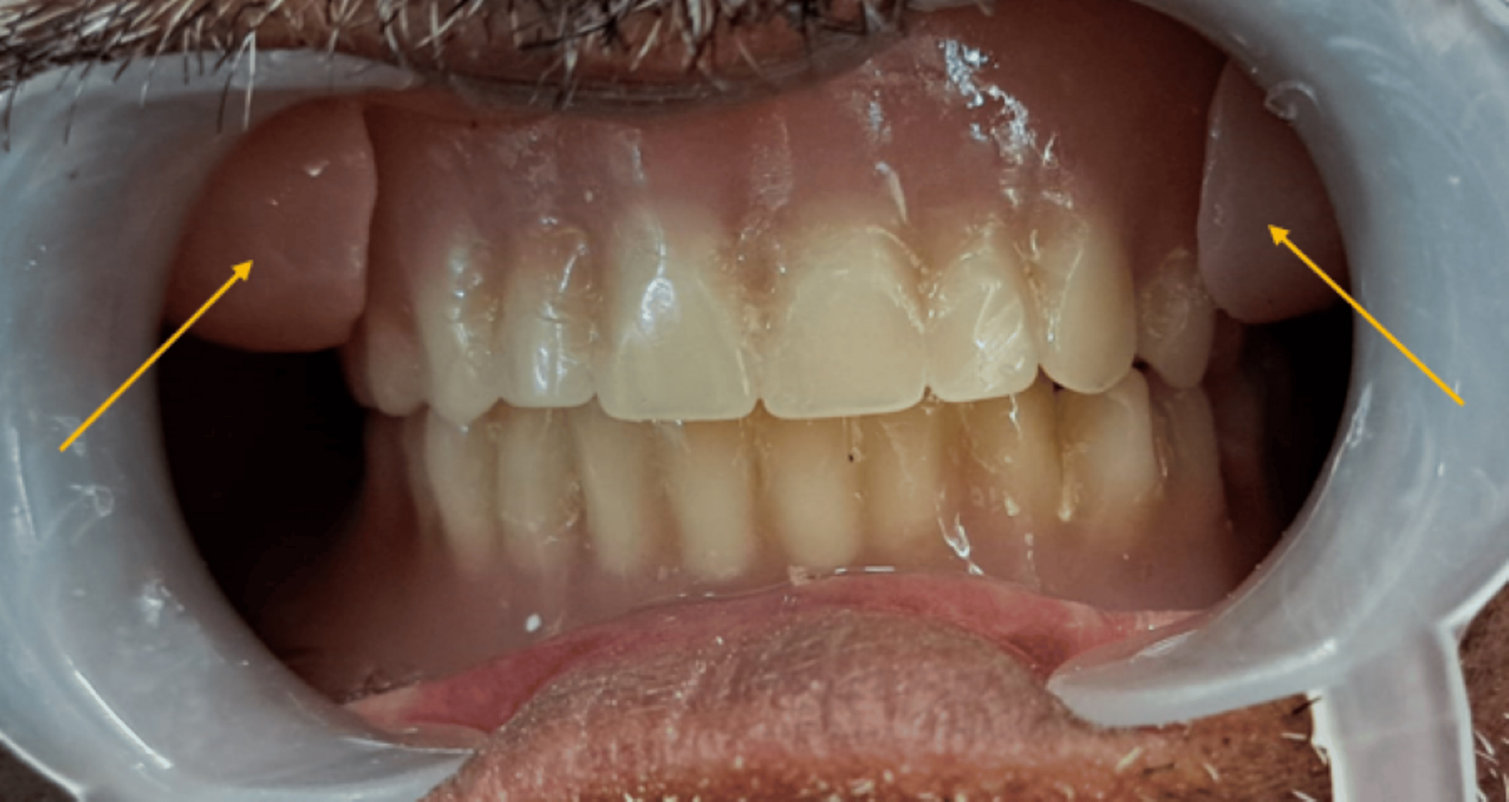
Post-insertion intraoral photograph. Arrow shows check plumper

The denture was fabricated using heat cure acrylic resin (DPI Heat Cure, Dental Products of India Ltd., Mumbai, India) by the traditional compression molding technique in accordance with the manufacturer's instructions. The processing of the detachable cheek plumper and maxillary complete denture prosthesis was done in two separate flasks, as shown in Figure 2. After flasking and dewaxing, packing was done. After retrieving the processed denture and detachable extensions of the cheek plumer, a 2mm deep and 5mm wide space was created using #8 no. round bur in the buccal flange area of the maxillary denture in the second premolar and molar regions for attachment of the press button. Matrix and Patrix components of press buttons were attached over the created space in the denture and cheek plumper, respectively, with the help of cold cure acrylic. The chances of press button dislodgment is less as it is attached with cold cure acrylic. A finished and polished denture was given to the patient, as shown in Figure 3. Post-insertion intraoral occlusion is seen in Figure 4. 

**Figure 5:**
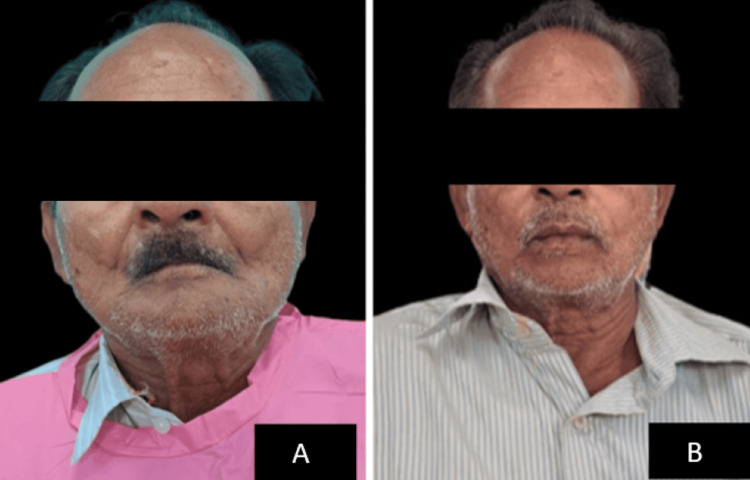
A: Before insertion of complete denture prosthesis; B: After insertion of complete denture prosthesis.

After insertion, the patient was recalled for follow-up after 24 hours, seven days, and one month to assess phonetics, function, comfort, and aesthetics. Throughout recall visits, the patient was extremely comfortable with the fit and comfort and did not exhibit any symptoms of muscular fatigue. Figure 5 shows the difference in the facial appearance of the patient before and after the insertion of a complete denture prosthesis with a detachable cheek plumper. 

## Discussion

Teeth loss, accompanied by sunken cheeks and lips, can have a devastating psychological impact on an individual. Wrinkles, creases, and sagging on the face can be minimized with proper support for the facial muscles. When teeth are missing, cheek plumpers can provide the muscles with sufficient support. The retention of the dentures is compromised by the unscrewable cheek plumper since it makes the dentures heavier and bigger. Moreover, people with microstomia cannot have their dentures inserted because of excessive mediolateral width in the cheek plumper area [5,6]. There are invasive and non-invasive methods for improving the appearance of drooping cheeks. Several techniques, including facial muscle injections of botulinum toxin (BOTOX) and reconstructive plastic surgery, are part of the invasive approach. A terrible plastic surgery operation is often not recommended for elderly individuals with a variety of medical conditions and can leave a scar after the procedure [6,7]. On the other hand, prosthetic techniques can be used to treat sunken cheeks non-invasively. It's a non-invasive technique.

Because of their larger size and weight, conventional cheek plumpers have significant limitations when it comes to stability and retention for patients wearing maxillary dentures. Due to its size, prolonged use may also result in muscle fatigue [8]. A detachable cheek plumper is a helpful device for preventing muscle fatigue and easing discomfort. It is easy to fabricate, use, and maintain hygiene. When microstomia is present, denture insertion and removal could be made easier with a detachable plumper prosthesis. A cheek plumper could be made using a variety of retentive aids, such as wire-retained cheek plumpers, customized ball attachments, friction lock attachments, stud attachments, dowl pins, and magnets [4,7-10]. The detachable cheek plumpers are easy to clean and maintain oral hygiene after eating. Although magnet-retained plumper prostheses have been employed, they have shown inadequate resilience to corrosion and a gradual loss of their magnetic properties [7,11,12]. Patients with metal allergies are not suitable for using magnets, which is a limitation of the magnet-retained cheek plumper. Patients should be made aware that the magnetic assembly is harmed by the magnetic field utilized in magnetic resonance imaging (MRI) exams. Patients have to take their dentures off for MRIs. Temperatures above 150°C should be avoided near the magnetic assembly [5]. The least expensive way to attach a cheek plumper to a denture is using a press-button attachment; clinical magnets are a costly alternative [13]. Thus, in this instance, a press-button cheek plumper is a thoughtful choice. Depending on the patient's skills and the thickness and height of the denture flange, clinicians can select the right attachment [14].

## Conclusions

A straightforward, non-invasive, and reasonably priced method of creating a cheek plumper is explained in this case study. Here, not only the patient's chewing efficiency was increased, but the patient also received psychological support from the improved appearance. Patients find it easier to wear removable cheek plumpers. The ability to carefully choose a cheek plumper case and attach the detachable cheek plumper with the correct technique is a skill.
